# Bronchial Fissuring: A Severe Complication of Arterial Aneurysm

**DOI:** 10.7759/cureus.43029

**Published:** 2023-08-06

**Authors:** Afaf Thouil, Meriem Rhazari, Habiba Alaoui, Khalid Serraj, Hatim Kouismi

**Affiliations:** 1 Laboratory of Research and Medical Sciences, Department of Respiratory Diseases, Faculty of Medicine and Pharmacy of Oujda, Mohammed VI University Hospital, Mohammed First University, Oujda, MAR; 2 Department of Pulmonology, Mohammed VI University Hospital, Oujda, MAR; 3 Laboratory of Immunohematology and Cellular Therapy, Department of Internal Medicine, Mohammed First University, Oujda, MAR; 4 Department of Internal Medicine, Mohammed First University, Oujda, MAR

**Keywords:** bipolar aphtosis, aphtosis, behçet, bronchial fissuring, aneurysm

## Abstract

Arterial aneurysms are rare and may occur in the context of Behçet's disease. The natural progression of these aneurysms can lead to an increase in their size and eventual rupture into the bronchi, causing life-threatening hemoptysis. We report a case of a subclavian artery aneurysm in a 30-year-old female patient with Behçet's disease who presented with moderate hemoptysis caused by a fistulized left subclavian artery aneurysm into the left main bronchus. The patient was treated with a bolus of corticosteroids followed by oral therapy, and six boluses of cyclophosphamide were scheduled. Unfortunately, the patient's condition deteriorated, and she died after her second cycle of cyclophosphamide following fulminant hemoptysis. The management of aneurysms in Behçet's disease is not standardized, but embolization appears to be the most promising conservative therapy.

## Introduction

Pulmonary arterial involvement in Behçet's disease is rare and may reveal the disease [1-3]. It is associated with a poor prognosis due to the spontaneous evolution of these aneurysms, which can lead to an increase in their size and rupture in the bronchi with fatal hemoptysis.

## Case presentation

**Figure 1:**
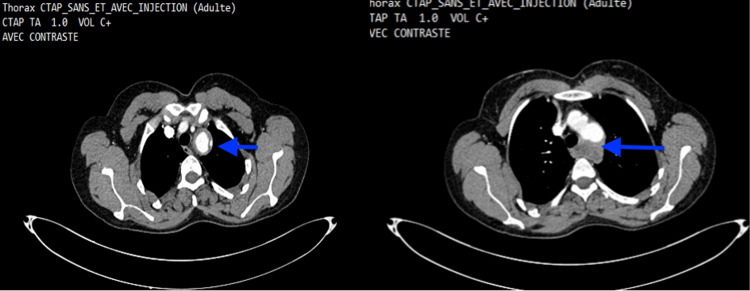
Partially thrombosed aneurysmal dilatations of the left subclavian artery origin (blue arrows) measuring 47 mm, causing mass effect on the trachea and left main bronchus.

A 30-year-old female patient presented with several episodes of oral and genital aphthosis. She had been experiencing moderate hemoptysis, dry cough, and exertional dyspnea for a month, with preservation of her general condition and apyrexia. The pleuropulmonary and cardiovascular examinations were normal. The cutaneous-mucosal examination revealed an oral aphthous ulcer and a genital scar. Thoracic and abdominopelvic CT scans with intravenous contrast were immediately requested due to a strong suspicion of pulmonary embolism, which showed partially thrombosed aneurysmal dilatations at the origin of the left subclavian artery measuring 47 mm, causing mass effect on the trachea and left main bronchus with wall irregularities, and a second partially thrombosed aneurysmal dilatation involving the origin of the left common iliac artery measuring 32 mm (Figure 1). The laboratory evaluation revealed hypochromic microcytic anemia with hemoglobin of 10 g/dL, ferritin of 37 ng/mL, C-reactive protein of 1 mg/dL, and sedimentation rate of 11 mm/h, with a positive pathergy test and a negative anti-neutrophil cytoplasmic antigen (ANCA).

**Figure 2:**
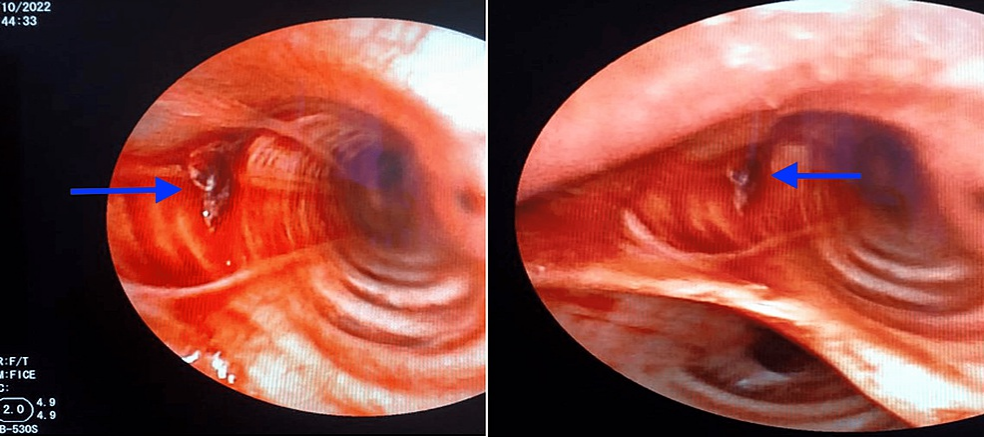
Bronchoscopy showed a fistula at the entrance of the left main bronchus with active bleeding (blue arrows).

The diagnosis of Behçet's disease with aneurysms of the left subclavian and common iliac arteries was made. Cardiac ultrasound was unremarkable with no intracavitary thrombus. Moreover, a bronchoscopy was performed, revealing a fistula at the entrance of the left main bronchus with active bleeding (Figure 2).

The ophthalmological examination did not reveal uveitis. The patient received a bolus of corticosteroids followed by oral therapy and six boluses of cyclophosphamide (600 mg/m^2^ every 15 days) associated with hemostatic treatment. Unfortunately, the patient's condition was marked by her death after her second cycle of cyclophosphamide following fulminant hemoptysis.

## Discussion

Arterial aneurysms can be indicative of Behçet's disease, as observed in our case. They generally affect the large pulmonary or lobar arterial trunks or, more rarely, segmental ones. They are often bilateral [1,2]. Thoracic involvement is rare (1-8%). It mainly includes superior vena cava thrombosis, pulmonary arterial aneurysms, and rarely pulmonary infarction and alveolar hemorrhage [4-6]. Increased blood flow, high internal pressure, degeneration, and weakness of the vascular wall contribute to the formation and progression of an aneurysm. The processes that lead to rupture are unknown, and the diameter is not the only risk factor for rupture [7].

Chest radiography shows rounded, hilar, or juxta-hilar opacities with clear boundaries. Sometimes, alveolar opacities blur due to secondary hemorrhagic alveolar filling from hemoptysis [3]. A chest CT scan with intravenous contrast confirms the vascular nature of aneurysmal opacities. It shows vascular wall thickening due to inflammatory and thrombotic phenomena [3-5]. Pulmonary angiography confirms the existence and extension of these aneurysms and often indicates partial thrombosis of the aneurysmal sac [4].

The treatment of these aneurysms is not standardized. Surgical treatment consists of resection of the aneurysm. Aneurysm regression has been achieved under corticosteroids, cyclophosphamide, or thalidomide. Aneurysm embolization is the most promising conservative therapeutic modality currently available [2].

The spontaneous evolution of these aneurysms leads to an increase in their size and their fissure in the bronchi, as is the case with our patient, which can be responsible for fatal hemoptysis. Sometimes they can retrocede spontaneously, perhaps due to intra-aneurysmal thrombosis. The prognosis mainly depends on the evolution of the aneurysms [4].

## Conclusions

In conclusion, this case highlights the rare but severe thoracic involvement of Behçet's disease, with the presence of aneurysms in the left subclavian and common iliac arteries causing mass effect on the trachea and left main bronchus, leading to fatal hemoptysis. The diagnosis of Behçet's disease can be challenging, but imaging studies such as chest CT scan with intravenous contrast can help confirm the presence of aneurysms. Treatment options for aneurysms in Behçet's disease are not standardized, but embolization appears to be the most promising conservative therapy. The prognosis of these aneurysms depends on their evolution and management.
